# A Subacute Presentation of Wunderlich Syndrome in a Young Woman: A Case Report

**DOI:** 10.7759/cureus.41385

**Published:** 2023-07-05

**Authors:** Julian D Cubillos, Daniel R Mejia, Edward E Cañas, Julian Serrano, Onofre Casas

**Affiliations:** 1 Emergency Medicine, Hospital Universitario San Ignacio, Bogota, COL; 2 Emergency Medicine, Pontificia Universidad Javeriana, Bogota, COL; 3 Medicine, Hospital Universitario San Ignacio, Bogota, COL; 4 Medicine, Pontificia Universidad Javeriana, Bogota, COL; 5 Emergency Physician, Pontificia Universidad Javeriana, Bogota, COL

**Keywords:** kidney diseases, perinephric hematoma, kidney tumors, renal angiomyolipoma, wunderlich syndrome

## Abstract

Wunderlich syndrome (WS) is a rare, potentially life-threatening medical condition characterized by spontaneous renal or perinephric hemorrhage occurring in the absence of known trauma. WS usually presents as Lenk's triad: acute flank pain, flank mass sensation, and hypovolemic shock; however, the presentation of this condition can vary in terms of symptom type and duration. We present the case of a 23-year-old previously healthy woman who consulted our emergency department with an unusual subacute form of presentation of WS (eight days of pain) due to an angiomyolipoma. Considering that the patient was clinically stable, a conservative approach with strict follow-up with serial computed tomography scans was taken.

## Introduction

Diagnosing abdominal pain is a challenge for the emergency physician because the range of differential diagnoses is wide. The onset, location, and time of evolution of abdominal pain can be helpful when discriminating among its many causes. Given the numerous etiologies of abdominal pain, clinicians often overlook infrequent diagnoses [1]; among those is Wunderlich syndrome (WS).

Carl Wunderlich first identified WS in 1856, citing the presence of a non-traumatic hemorrhage in the subcapsular and perirenal spaces [2]. This condition is more frequent in men; it appears at an average age of 46 years, with most cases occurring between the ages of 30 and 60 years, and usually presents with the Lenk's triad, comprised of sudden flank pain, flank mass sensation, and hypovolemic shock. However, this triad occurs in approximately 20% of WS patients. Although WS was described more than 100 years ago, only 165 cases were reported from 1985 to 1999, and this number was further reduced to 102 reported cases between 2000 and 2016 [3-5] and to 41 cases between 2016 and 2022, indicating its rarity.

As mentioned above, WS usually presents as an acute condition, but it manifests less often in a more insidious and progressive manner. Given the rarity of WS and the fact that it can be life-threatening, it is important for the emergency physician to recognize this entity since it is usually confused with other frequent pathologies, such as renal colic. Imaging techniques such as computed tomography (CT) urograms can be helpful for diagnosing this potentially fatal condition and ruling out differential diagnoses [6]. Thus, we present the case of a patient with an atypical, insidious presentation of WS to provide the clinician with elements to recognize this rare condition.

## Case presentation

A 23-year-old previously healthy female presented to the emergency department with a four-hour course of symptoms consisting of left lumbar pain with an intensity of 5/10 radiating to the ipsilateral flank. On review of systems, she referred to three emetic episodes, leukorrhea, and dyspareunia during the last two weeks. On admission, she was afebrile, had a blood pressure of 115/85 mmHg, a heart rate of 82 beats per minute, a respiratory rate of 19 breaths per minute, an oxygen saturation of 94%, and a temperature of 36.5 °C. The abdomen was soft but tender on both left quadrants, but without signs of peritoneal irritation; the patient had no costovertebral angle tenderness, and no palpable masses were felt. There were no other relevant findings from the physical examination. Based on the clinical features, renal calculi were suspected. Analgesic management was initiated.

Due to the suspicion of urolithiasis, a hemogram, renal function, and partial urine tests were requested in order to assess complications of urolithiasis such as anemia or hematuria, assess kidney function to detect any impairment due to obstruction, and rule out some differential diagnoses that may mimic renal colic, such as a urinary tract infection. The paraclinical test results on admission are shown in Table 1.

**Table 1:** Paraclinical tests BUN: blood urea nitrogen, INR: international normalized ratio

Variable	First hospitalization	Second hospitalization	Reference values
Hemoglobin	14.4 g/dL	12.3 g/dL	12–16
Hematocrit	41.8%	38.4%	36–44
Platelets	163,800 µ/L	338,800 µ/L	150,000–450,000
Qualitative pregnancy test	Negative	-	
Creatinine	0.65 mg/dL	0.56 mg/dL	0.7–1.3
BUN	18.3 mg/dL	18 mg/dL	6–24
Prothrombin time	-	11.5 seconds	10–13
Thromboplastin time	-	32.7 seconds	25–35
INR	-	1	
Partial urine test			
Density	1.027	1.025	1.005–1.030
pH	5.5	5.5	4.6–8
Leucocytes	Negative	Negative	
Nitrites	Negative	Negative	
Proteins	Negative	Negative	
Glucose	Normal	Normal	
Ketone bodies	10 mg/dL	10 mg/dL	<20
Urinary sediment			
Red blood cells	1 HPF	1 HPF	<5
Leukocytes	2 HPF	1 HPF	<10
Squamous cells	2 HPF	2 HPF	<15
Bacteria	+	+	
Urine gram	No germs observed	No germs observed	

**Figure 1:**
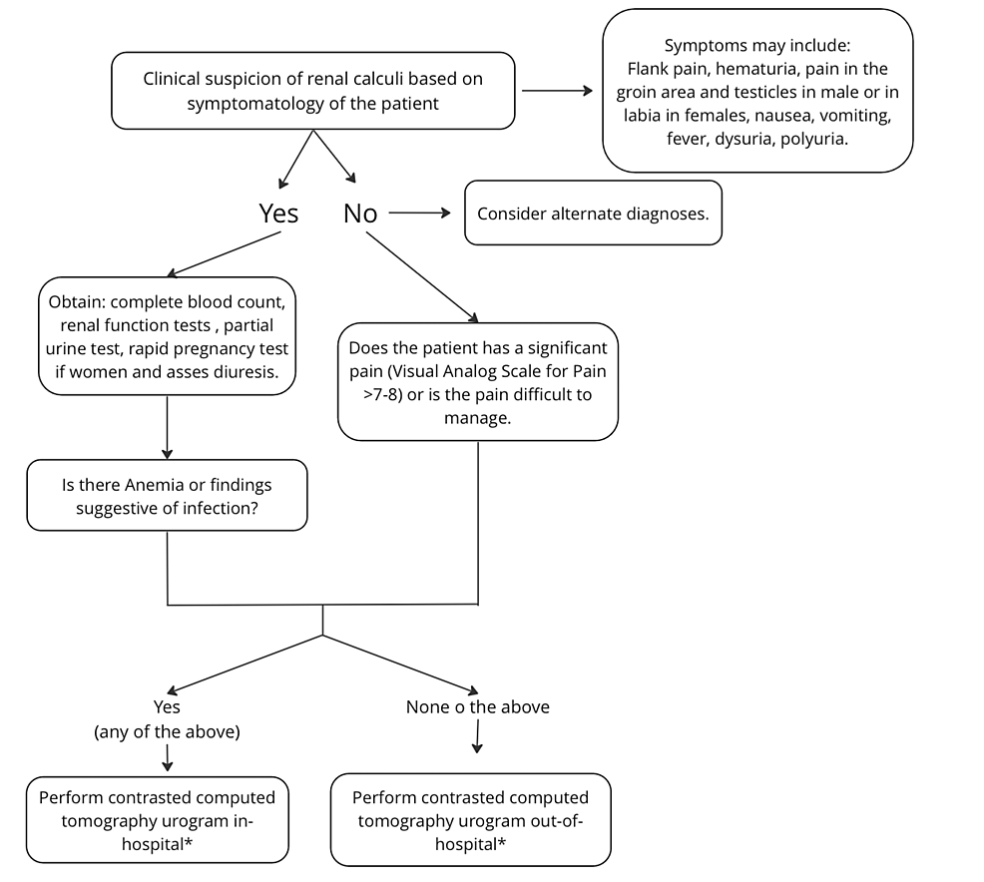
An initial diagnostic approach for the patient with suspicion of renal calculi at our emergency department. *Always asses renal function before taking a contrasted CT image.

Given the normal results of the aforementioned studies and the referred leukorrhea and dyspareunia, an evaluation by the gyneco-obstetrics service was requested. They reported a normal appearance of the external genitalia with no signs of inflammation, lesions, or abnormal discharge, and the gynecological speculoscopy did not show any significant abnormalities of the vaginal walls or cervix. A transvaginal pelvic ultrasound was performed, and no adnexal lesions, masses, or free fluid were found. Based on the anamnesis and physical examination, a diagnosis of fungal vaginitis was made, and the patient was discharged with clotrimazole, indicating a possible functional etiology of her abdominal pain. Although renal calculi could not be ruled out on the basis of the patient's symptoms, given the patient's adequate symptomatic control (pain) and the fact that the initial paraclinics were normal, it was decided to order an ambulatory computed tomography urogram. Our institution does not perform ultrasonography to diagnose renal calculi, taking into account its sensitivity and specificity (45% and 88%, respectively) for calculi >3 mm, but rather performs a computed tomography urogram directly based on the severity of symptoms or if there is any paraclinical abnormality, considering that it is the gold standard for diagnosing renal calculi [7]. The diagnostic approach to renal calculi used at the emergency department at our institution is shown in Figure 1.

**Figure 2:**
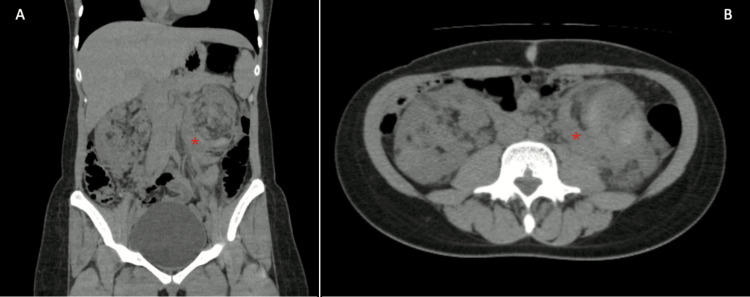
Contrasted computed tomography urogram. (A) and (B) Frontal and axial computed tomography urogram showing an increased size of both kidneys with severe distortion of their architecture architecture due to focal lesions, with fat density compatible with angiomyolipomas, with a subacute left perirenal hematoma (marked with: *).

Eight days after her discharge, she reconsulted because of persistent pain in the left flank, which increased in intensity within the last 24 hours prior to the consult, reaching an intensity of 9/10. There was associated nausea and occasional emesis, with no fever, urinary or gynecologic abnormalities, or other associated symptoms. On admission, she had a blood pressure of 125/90 mmHg, a heart rate of 80 beats per minute, a respiratory rate of 20 breaths per minute, an oxygen saturation of 93%, and a temperature of 36.5 °C. Abdominal examination revealed tenderness on palpation of the left quadrants, but without signs of peritoneal irritation; the patient had no costovertebral angle tenderness, and no palpable masses were felt. There were no other relevant findings from the physical examination. Based on the clinical features, urolithiasis was suspected, and given the severity of her symptoms (pain), a contrasted computed tomography urogram (Figure 2) was requested. The patient did not perform the computed tomography urogram requested in her last consultation at the emergency department. Due to the suspicion of urolithiasis, a hemogram, renal function tests, and a partial urine test were requested. The paraclinical test results on readmission, which were all normal, are also shown in Table 1.

Based on imaging, a diagnosis of WS secondary to an angiomyolipoma was made since the patient denied recent trauma. Coagulation tests were requested at the time of diagnosis to rule out a concomitant coagulation disorder with the tumor that could explain the bleeding; the results of the tests are shown in Table 1, which were normal. An evaluation by the urology service was then requested, and they determined that due to the high risk of intraoperative mortality, given the anatomopathological characteristics of the angiomyolipoma and considering the patient is clinically stable, expectant management should be done, with serial follow-ups with renal function and imaging tests. To date, 18 months after her discharge from the emergency department, the patient has not presented with rebleeding or deterioration of renal function; therefore, she continues in strict follow-up and has not required surgery.

## Discussion

WS is a rare condition that can be potentially fatal; therefore, it is essential that emergency physicians know how to diagnose and treat this condition. Computed tomography is the imaging technique of choice for the diagnosis and follow-up of this type of patient because, as in our case, it allowed us to diagnose the characteristic bleeding and establish an etiology. For emergency physicians, other tools such as bedside ultrasonography are of special relevance in evaluating WS patients with signs of hypovolemic shock because they allow the evaluation of the hemodynamic status of the patient, specifically on parameters such as the vena cava index and time-to-integral velocity (VTI), which have a direct relationship with the prognosis of these patients [8-10].

The etiologies of WS can be divided into oncologic, which are the more frequent, accounting for 63% of cases, and non-oncologic. Among oncologic causes, angiomyolipoma is the most frequent, followed by renal cell carcinoma. Among non-oncologic causes, the most frequent etiologies are vascular diseases, such as vasculitis, renal artery aneurysms, arteriovenous malformations, and coagulation disorders, which include a diverse group of conditions ranging from anticoagulant intakes to coagulopathy due to severe hepatic failure. Other less frequent causes include hydronephrosis, renal cysts, lithiasis, and adrenal gland disorders [10]. WS is frequently found in conjunction with hypertension and atherosclerosis [11].

Regarding the clinical presentation, our case had several peculiarities with respect to what is described in the literature. First, our patient was female, and WS is more common in males. In addition, the patient was 23 years old at the time of her consultation, marking an early age of presentation since the average age is 46 years. There is limited information available on why WS appears more frequently at older ages and in males. However, some studies suggest that this association is due to the higher prevalence of risk factors such as smoking, hypertension, atherosclerosis, and neoplasms in older adults and males [11,12]. Finally, the instauration of symptoms is usually fast, and our patient had an insidious and progressive evolution of eight days [4].

Paraclinical testing in WS is crucial because it helps in the evaluation and differential diagnosis of the condition. Paraclinical tests such as the complete blood count, renal function tests, coagulation profile, and urinalysis can provide valuable information about the patient's overall health status, helping to assess the severity of the condition, kidney function, and the presence of any related abnormalities. For example, a complete blood count can help determine the severity of the hemorrhage since anemia can indicate that the patient has lost a significant amount of blood. A low platelet count (thrombocytopenia) can indicate that the patient has a bleeding disorder, helping to identify the etiology of WS [8,12].

Diagnostic imaging is important in WS because it can help identify the underlying cause of the hemorrhage. CT scans are considered the most relevant imaging test for the diagnosis of WS, as they provide detailed and accurate imaging of the renal and perinephric areas and help establish the underlying cause of the syndrome. In some cases, magnetic resonance imaging (MRI) can be used if CT provides limited or inconclusive information and fails to identify the etiology of WS, since MRI scans can provide more detailed information about the underlying cause of the hemorrhage than CT scans. Renal angiography can be used in selected cases to diagnose the underlying cause (such as aneurysms or other vascular abnormalities) and control active bleeding, potentially avoiding unnecessary surgical interventions. Ultrasound may detect perinephric hematomas, but its sensitivity is much lower than that of CT and MRI. In most patients, the etiology cannot be detected using this imaging technique alone. The timing of diagnostic imaging is important in WS, as it helps guide treatment by identifying the cause of the syndrome. However, if the patient is in shock or has other life-threatening symptoms, imaging may be delayed until the patient's condition has stabilized [8,12-14].

Treatment depends on the hemodynamic status of the patient. In cases of hemodynamic instability, an urgent radical nephrectomy or hemostasis by interventional radiology should be performed. If the patient is hemodynamically stable, a conservative approach should be chosen, and follow-up with serial CT scans should be performed to monitor bleeding and determine the cause of the hemorrhage before elective resolutive surgery, if applicable, with low morbidity and mortality rates. This strategy was performed on our patient, who showed adequate evolution as she did not present with any complications during her follow-up [10,15].

## Conclusions

This case report contributes to the medical literature by presenting an unusual subacute presentation of WS in a young female patient with symptoms that had been present for eight days. This case highlights the importance of adequate medical examinations and imaging techniques in diagnosing this potentially fatal condition.
